# How to quantify magic spots - a brief overview of (p)ppGpp detection and quantitation methods

**DOI:** 10.3389/fmolb.2025.1574135

**Published:** 2025-03-25

**Authors:** Filip Gąsior, Wiktoria Klasa, Katarzyna Potrykus

**Affiliations:** Department of Bacterial Molecular Genetics, Faculty of Biology, University of Gdansk, Gdańsk, Poland

**Keywords:** (p)ppGpp, (p)ppApp, alarmones, stringent response, stress response, *in vivo* and *in vitro* quantification, nucleotides, chromatography

## Abstract

Guanosine tetra- and penta-phosphates, collectively known as (p)ppGpp, are well-known second messengers of cellular stress responses in bacteria and plants. Their intracellular concentration is tightly regulated and can vary widely–from undetectable levels under optimal growth conditions, through intermediate concentrations, to extremely high levels that match or even exceed GTP concentrations when cells are exposed to severe stress. Importantly, the effects exerted by (p)ppGpp are often concentration-dependent, making their quantitative analysis a crucial aspect of studying cellular responses to stress. To gain a deeper understanding of the regulatory mechanisms associated with (p)ppGpp, it is essential to monitor its accumulation *in vivo* and conduct detailed molecular studies *in vitro*. Various methods have been developed for detecting and quantifying (p)ppGpp, enabling researchers to track its levels in living cells and analyse its function under controlled laboratory conditions. In this work, we provide an overview of the available techniques for (p)ppGpp detection and quantification. We present their advantages, limitations, and potential applications in research on metabolic regulation and cellular stress responses.

## 1 Introduction

All living organisms are exposed to stress, caused by biotic or abiotic conditions, and in order to survive they must quickly adjust their metabolism which is often correlated with rapid changes in gene expression. Among different modes of response to stress, one of the best known is the stringent response in bacteria, characterized by rapid synthesis of guanosine nucleotide derivatives, namely, guanosine penta- and tetraphosphate (ppGpp and pppGpp, jointly abbreviated as (p)ppGpp). These nucleotides were initially called “magic spots”, and are often referred to as alarmones, because they alarm the cell about changing environmental conditions. Under stress their levels can rise about 100-fold above basal level (reviewed in Steinchen et al., 2020). In *Escherichia coli* this means reaching equimolar concentration to GTP (Potrykus and Cashel, 2016), while in *Bacillus subtilis* (p)ppGpp concentration was shown to exceed that of GTP, which drops dramatically upon alarmone production (Lopez et al., 1981; Krásný and Gourse, 2004; Kriel et al., 2012). In addition to ppGpp and pppGpp, another guanosine derivative – pGpp, was shown to exist in Firmicutes (Bacillota) and other Gram-positive bacteria, and its role in bacterial cells is intensely investigated (Gaca et al., 2015; Yang et al., 2020).

All in all, the (p)ppGpp alarmones are responsible for prokaryotic cell survival under such stresses as amino acid starvation, nitrogen-, iron-, fatty acid-, phosphate- and carbon-limitation, acidic shock, and they were also shown to be required for bacterial pathogenicity (Potrykus and Cashel, 2008; Dalebroux et al., 2010; Irving et al., 2021; Urwin et al., 2024). In addition, (p)ppGpp has been shown to exist in plants and it seems to enhance plant survival under increased salinity, as well as to play a role in photosynthesis (Mehrez et al., 2023). Intriguingly, a (p)ppGpp degrading enzyme (called Mesh1) was discovered to exist in metazoa, such as fruit flies, mice and humans (Sun et al., 2010), although it was later shown to also possess a NADPH hydrolysis activity and thus play a role in ferroptosis (Ding et al., 2020; Chi and Zhou, 2023). No (p)ppGpp synthetase has been discovered in metazoa to date, however, there is one report where ppGpp was detected in germ-free *Drosophila melanogaster* and several eukaryotic cell lines (Ito et al., 2020).

Overall, the (p)ppGpp level reflects on cellular physiology and its elevation signifies the cells undergo some kind of stress, although the alarmone levels may not necessarily need to reach the maximum concentration in order to exert regulatory effects (see Steinchen et al., 2020). Thus dissecting specific mechanisms of action necessitates (p)ppGpp detection and precise quantitation, and this is true not only for prokaryotic but also for eukaryotic cells (e.g., plants). Besides *in vivo* studies that give a generalized glimpse at cell physiology, (p)ppGpp *in vitro* studies are also important since they are pivotal to determine molecular basis of alarmone metabolism, such as their synthesis and degradation. This is especially important in the era of increased bacterial antibiotic resistance, as using novel drugs to disrupt such processes is an attractive possibility. Hence, here we present a comprehensive mini-review on different approaches to determining (p)ppGpp levels, both for *in vitro* and *in vivo* applications.

## 2 (p)ppGpp detection and quantitation

As mentioned above, detection and quantitation of (p)ppGpp alarmones is essential for understanding their roles in stress responses, yet the task is methodologically challenging due to the dynamic nature of their metabolism and sometimes low intracellular concentrations. The chemical properties of (p)ppGpp, such as multiple phosphate groups and its structural similarity to other abundant nucleotides, e.g., ATP and GTP, also significantly complicate its analysis. These aspects necessitate the use of highly specific detection methods to prevent misidentification. Below we present the most common techniques used for (p)ppGpp detection and quantitation.

### 2.1 Thin layer chromatography (TLC)

Thin-layer chromatography (TLC) is a versatile analytical technique used to separate compounds based on their differing affinities to a stationary phase and a mobile phase solvent, and enables both qualitative and quantitative analysis. In addition, it offers such advantages as low cost, simplicity, and minimal sample preparation, making it a popular choice in biochemical and molecular biology studies. For separating nucleotides, plates coated with polyethyleneimine-cellulose (PEI-cellulose) are employed (Randerath and Randerath, 1964).

The TLC method of detection was initially used by Drs. Cashel and Gallant when they first discovered (p)ppGpp, which they called “magic spots” at that time because their chemical nature was unknown (Cashel and Gallant, 1969). In that seminal paper, *in vivo* labeling with inorganic P^32^-phosphate was employed, followed by nucleotide extraction with formic acid and sample resolution in two dimensions (with LiCl step gradient in the first dimension, and sodium formate step gradient in the second dimension). This method was later improved by employing different buffer systems, e.g., formate gradient in the first dimension, followed by 0.85 M KH_2_PO_4_ (pH 3.4) in the second dimension (Cashel et al., 1969) or 4 M formic acid combined with 1 M LiCl in the first dimension, followed by 1.5 M KH_2_PO_4_ (with no pH adjustment) in the second dimension (Nishino et al., 1979). These conditions allow for efficient separation of all NTPs and dNTPs, as well as (p)ppGpp and (p)ppApp (adenosine derivatives homologous to (p)ppGpp which were first discovered in *B. subtilis* (Rhaese et al., 1977) and are lately gaining much attention (e.g., Sobala et al., 2019; Anderson et al., 2021; Ahmad et al., 2023)).

Over the years, the TLC protocol for (p)ppGpp detection and quantification has been simplified to be run only in one direction, very often with 1 M or 1.5 M KH_2_PO_4_ (pH 3.4) as the solvent (e.g., Kuroda et al., 1997; Bruhn-Olszewska et al., 2018; Sobala et al., 2019). It should be stressed, however, that under these conditions ppGpp comigrates with pppApp, and similarly ppApp comigrates with GDP (Sobala et al., 2019) and thus care should be taken if its suspected that both (p)ppGpp and (p)ppApp may be present in a given sample. In such a case, two-dimensional TLC is recommended (Sobala et al., 2019). On the other hand, to separate pGpp from GTP, 0.75 M KH_2_PO_4_ (pH 3.4) was shown to work well (Gaca et al., 2015).

How to visualize and quantify (p)ppGpp on TLC plates? It depends on the approach used. If *in vivo* labeling is employed as in the original studies (with P^32^- or P^33^-orthophosphate), the plates are run and autoradiograms are developed either by exposure with X-ray film or phosphor-storage screens, and followed by densitometry (see e.g., Mechold et al., 2013 for details). If *in vitro* methods are employed, i.e., pure (p)ppGpp nucleotides are investigated, then the alarmones can be detected under UV light (λ = 254nm; e.g., Sobala et al., 2019). Alternatively, if labelled alarmones are examined *in vitro*, detection can be carried out either by autoradiography (if P^32^- or P^33^- labeled; e.g., Mechold et al., 2013; Sobala et al., 2019) or by cutting out relevant spots from TLC plates after the run is complete and using liquid scintillation counting (if labeling with H^3^, e.g., Manav et al., 2018).

### 2.2 High-performance liquid chromatography (HPLC) with or without mass spectrometry

The use of high performance liquid chromatography (HPLC) to separate nucleosides and nucleotides from *in vitro* obtained samples dates back to the 1940s with cation-exchange chromatography (Elmore, 1948; Cohn, 1949). However, in order to analyse nucleotide pools in bacterial cells, this approach has been later improved by using strong anion-exchange (SAX) chromatography (Payne and Ames, 1982). This method had originally allowed to separate and assess nucleotide levels, including ppGpp and pppGpp, in *Salmonella typhimurium* LT7 cell extracts, although it required a rapid extraction process, which indirectly determined the amount of components in the analysed sample (Payne and Ames, 1982; Fisher et al., 1982). Later on, this method was also shown to work for detecting (p)ppApp in *Pseudomonas aeruginosa* extracts, however, pppApp could not be separated from ppGpp (Ahmad et al., 2019). Thus, care should be taken if both types of nucleotides are expected in a given sample.

On the other hand, in comparison to SAX-HPLC, the ion-pair reverse phase (IPRP) chromatography was shown to significantly improve resolution of different nucleotides in a given sample (Payne and Ames, 1982). Also, the C18 columns were used successfully not just for resolving nucleotide mixtures but also for detecting ppGpp in bacterial extracts, e.g., *E. coli* (Vinella et al., 1992), and *Vibrio* sp. (Flärdh et al., 1994). However, it is suggested that since SAX better resolves ppGpp and pppGpp, while IPRP is better for resolving all other nucleotides (IPRP is inefficient in detecting pppGpp), both approaches should be used in parallel to obtain a full picture of the nucleotide and alarmone pools in the cell (Buckstein et al., 2008; Varik et al., 2017).

Despite the many advantages of anion-exchange and ion-pair reverse phase chromatography in the separation of nucleotides, these techniques are not ideal and have their limitations. In the case of IPRP, the reagents used may potentially damage the column, while for SAX it is contamination of the system with high salt concentrations which can lead to time consuming equilibration of the system after analysis is complete (Jin et al., 2018). One of the alternatives is hydrophilic interaction liquid chromatography (HILIC), in which elution is based on the competition between the phosphate groups of nucleotides and phosphate salts in the running buffer for binding to the amide groups on the column (Jin et al., 2018). This approach allowed for successful quantitation of ppGpp in the *Hameatococcus pluvialis* alga (Jin et al., 2018).

Combining HPLC with mas spectrometry (MS) may yield even better quantitative results. Due to the high content of organic substances used in the HILIC mobile phase, known to improve electrospray evaporation, the MS signal of evaluated nucleotides is greatly enhanced when compared to MS following other HPLC techniques (Zborníková et al., 2019). The combination of HILIC with MS was shown to successfully separate ppGpp from pppGpp in *E. coli* K12 cell extracts (Zborníková et al., 2019), or ppApp from pppApp for *P*. *aeruginosa* extracts (Ahmad et al., 2019). On the other hand, the IPRP technique coupled with MS was shown to be successful in detection and quantification of (p)ppGpp, (p)ppApp, and other nucleotides from *B. subtilis* (Fung et al., 2020). However, it is important to follow a specific protocol of nucleotide extraction from bacterial samples (Fung et al., 2020).

The above described HPLC methods work well for bacteria and algae, however, in the case of plants where the (p)ppGpp level is lower, it was necessary to introduce modifications of the sample analysis technique to enable its detection without the need to extract it from large amounts of plant tissue. Initially, studies conducted to analyse the (p)ppGpp level in plants were based on nucleotide solid-phase extraction (SPE) on a cellulose column, followed by SAX chromatography, thanks to which the presence of this alarmone in plants was shown for the first time (Takahashi et al., 2004). However, the minimum plant material allowing for such detection was rather large (∼20 g). The solution to this problem was the use of SPE with a combination of two resins mixed together: a weak-basic anion-exchange resin (to bind ppGpp as an anion) and a reversed-phase mixed-mode resin (to allow separation of ppGpp based on its hydrophobicity) (Ihara et al., 2015). This was followed by IPRP coupled with a tandem quadrupole mass spectrometry (UPLC-ESI-qMS/MS), and allowed for ppGpp detection in *Arabidopsis thaliana* in as little as 100 mg of plant tissue (Ihara et al., 2015). Still, to be accurate, the UPLC-ESI-qMS/MS method requires that the investigated sample be divided into two parts assayed in parallel, and a (p)ppGpp standard should be added to one of them in order to assess for the losses incurred during the procedure, e.g., during nucleotide extraction (Ihara et al., 2015). A solution to this problem is to introduce stable isotope-labelled standards, ^13^C-GTP and ^13^C-ppGpp, enabling compensation for nucleotide losses from plant tissues by measuring the concentrations of these standards before and after the extraction process in undivided sample (Bartoli et al., 2020). A similar approach was employed to detect ppGpp in metazoan cells, except that EDTA was added to the SPE buffer in order to enhance ppGpp concentration in samples applied to UPLC-ESI-qMS/MS (Ito et al., 2020).

### 2.3 Capillary electrophoresis (CE)

Capillary electrophoresis coupled with MS, commonly used in metabolomic approaches to study bacterial extracts, has been also successful in detecting ppGpp (Ooga et al., 2009; Takeuchi et al., 2012). However, pppGpp levels were below detection in these assays. On the other hand CE coupled with UV detection has led to detection of ppGpp, pppGpp, ppApp and pppApp, in defined nucleotide mixes containing all of these nucleotide derivates at once (Haas et al., 2020). Yet, for quantitative purposes the CE-MS method has been further improved to employ fully N^15^-labeled (p)ppGpp standards serving as internal references, which was successfully used to quantify ppGpp in as little as 150 mg of the plant material (Qiu et al., 2023).

### 2.4 Fluorescent and colorimetric detection techniques

One of the first approaches to determine (p)ppGpp concentrations by non-chromatography techniques employed PyDPA, a fluorescent chemosensor based on pyrene (pyrene-bis(Zn^2+^-dipicolylamine); Rhee et al., 2008). This dye was shown to produce fluorescence upon selective binding to (p)ppGpp’s pyrophosphate groups. The report concerning this technique was focused on *in vitro* ppGpp accumulation assessment (Rhee et al., 2008), but whether this method would be applicable to cellular extracts is unknown. In addition, although ppGpp yielded higher fluorescence signal than pppGpp, it would not be possible to distinguish between two of them in a mixture. Also, even though it could be suspected that (p)ppApp would also interact with PyDPA, such studies have not been conducted to date.

Another examples of non-chromatographic technique for (p)ppGpp detection is the use of RNA-based fluorescent sensors which is an innovative solution for real-time imaging of alarmones within living cells. This technique, described by (Sun et al., 2021), relies on an aptamer whose proper folding depends on (p)ppGpp. In turn, this incurs a proper folding of a fluorogenic RNA, which is located between the aptamer domains. Expression of such an aptamer - fluorogenic RNA fusion was then induced in *E. coli* cells, where in the presence of a specific dye (DFHBI-1T), the fluorescence signal was shown to be emitted in a (p)ppGpp dose-dependent manner. Although very attractive, the aptamer used could not differentiate between ppGpp and pppGpp (Sun et al., 2021). However, this technique is very promising, as bioinformatics analyses suggest that over one hundred aptamers could potentially bind (p)ppGpp across diverse bacterial species (Sherlock et al., 2018), and in fact there is a class of aptamers that distinguish between the two alarmones (Jagodnik et al., 2023).

More recently, it has been demonstrated that (p)ppGpp and its analogues (pGpp and ppGp) can be directly detected *in vitro* by using malachite green (MG) assay (Schicketanz et al., 2024). This assay has been originally devised to assess free orthophosphate concentrations (Itaya and Ui, 1966) and readings can be simply taken with a use of spectrophotometer. Thus, this technique offers simplicity combined with cost-effectiveness, although it cannot be used to differentiate between (p)ppGpp and its analogues in a mixture or be used to detect them in biological samples because MG may interact with other cellular components. Still, it has been used to successfully screen for SpoT (a (p)ppGpp synthesizing enzyme) inhibitors *in vitro* (Schicketanz et al., 2024) and may be imagined to work well for other *in vitro* applications, such as determination of enzyme kinetics for proteins involved in (p)ppGpp metabolism. Quite interestingly, the MG assay has been also used to detect NADPH hydrolysis by MESH1 (Ding et al., 2020).

## 3 Discussion

The techniques described above are examples of the methods most commonly used for (p)ppGpp detection and quantitation. We hope this brief overview will provide a useful starting point for those looking to quantify (p)ppGpp alarmone levels, whether *in vitro* or *in vivo*.

Each of the methods described has their advantages and limitations (summarized in Table 1). For example, even though TLC seems the simplest and the most robust for assessing many samples at once, not everyone may have access to a radioisotope laboratory or a phosphorimager, if *in vivo* analysis is required. The same is true for HPLC, CE and MS techniques, that require specialized equipment while do not always offer the expected alarmone separation. On the other hand, such techniques as the colorimetric MG assay do not require expensive equipment, however, the major limitation here is that pure alarmone preps must be employed since this method will also detect (p)ppGpp analogs, such as ppGp (Schicketanz et al., 2024), which is a common product of ppGpp’s spontaneous hydrolysis. Thus, even though it is a simple method, it should be in the good laboratory practice that the chemical nature of the alarmone under investigation be verified at the beginning of the experiment by a complementary method, such as non-radiolabeled TLC or HPLC. This is true for all methods that cannot differentiate between ppGpp, pppGpp and their analogues in a solution.

**TABLE 1:** Advantages and limitations of methods commonly applied for (p)ppGpp detection and quantitation. HPLC–high performance liquid chromatography, LC–liquid chromatography.

Technique	Advantages	Limitations
Thin-layer chromatography (TLC)	- Qualitative and quantitative determination of (p)ppGpp and (p)ppApp from *in vitro* and *in vivo* obtained samples (Cashel et al., 1969; Nishino et al., 1979; Kuroda et al., 1997; Mechold et al., 2013; Sobala et al., 2019) - No specialized equipment required	- Care should be taken to optimize resolution of nucleotides investigated (Cashel et al., 1969; Mechold et al., 2013; Sobala et al., 2019) - Access to radioisotope laboratory is required if assaying radiolabeled (p)ppGpp samples
Isocratic strong anion exchange - HPLC (SAX - HPLC)	- (p)ppGpp quantification in *in vitro* and *in vivo* obtained samples (Payne and Ames, 1982; Fisher et al., 1982)	- Limited resolution for other nucleotides when assessing *in vivo* samples (Payne and Ames, 1982; Varik et al., 2017) - Specialized equipment required - High salt concentrations used may require long system recalibration times (Jin et al., 2018)
Ion-Pair with C18 Reverse-Phase HPLC (IPRP - HPLC)	- ppGpp quantification in *in vitro* and *in vivo* obtained samples (Vinella et al., 1992; Flärdh et al., 1994) - Good resolution of other nucleotides even for *in vivo* samples (Varik et al., 2017)	- Limited resolution for pppGpp *in vivo* samples (Varik et al., 2017) - Specialized equipment required - Reagents used may lead to system and column damage, and may require long system recalibration times (Jin et al., 2018)
Hydrophilic Interaction LC (HILIC)	- Rapid detection of nucleotides, quantification of even small amounts of ppGpp for *in vivo* samples (Jin et al., 2018)	- Different columns have different retention times and even retention order for the same nucleotides (Jin et al., 2018) - Specialized equipment required
Liquid chromatography followed by mass spectrometry (LC-MS)	- Quantification of (p)ppGpp and (p)ppApp in *in vitro* and *in vivo* samples (Fung et al., 2020)	-There may be extraction challenges for *in vivo* samples (Fung et al., 2020; Zborníková et al., 2019) - Specialized equipment required
Ultra-performance LC coupled with a tandem quadrupole mass spectrometer (UPLC-ESI-qMS/MS)	- Quantification of ppGpp in plants (Ihara et al., 2015; Bartoli et al., 2020)	- Requires isotope-labelled standards for precise quantification of ppGpp (Bartoli et al., 2020) - Specialized equipment required
Capillary Electrophoresis (CE)	- Detection of (p)ppGpp and (p)ppApp in complex nucleotide mixes in one run (Haas et al., 2020) - Quantification of ppGpp in plants (Qiu et al., 2023)	- Requires isotope-labelled standards for precise quantification of ppGpp (Qiu et al., 2023) - Specialized equipment required
Pyrene-based chemosensor (PyDPA) assay	- Real-time *in vitro* detection of (p)ppGpp (Rhee et al., 2008) - No specialized equipment required	- Does not differentiate between pppGpp and ppGpp (Rhee et al., 2008) - Decrease in fluorescence at (p)ppGpp concentrations above a certain threshold (Rhee et al., 2008)
RNA-based live-cell imaging (RNA Fluorescent Sensor)	*-* *In vivo* real-time imaging (Sun et al., 2021)	- Different variants of RNA sensors have different detection ranges and response rates (Sun et al., 2021)
Malachite Green (MG) assay	- Quantification of (p)ppGpp from *in vitro* samples (Schicketanz et al., 2024) *-* No specialized equipment required	- Not appropriate for *in vivo* samples (Schicketanz et al., 2024) - Does not differentiate between pppGpp, ppGpp and their analogues, nor free phosphate (Itaya and Ui, 1966; Schicketanz et al., 2024)

It should be also noted that all of these methods require (p)ppGpp standards to be run in parallel with the samples analyzed. These can be obtained by in-house biochemical synthesis (e.g., Mechold et al., 2013; Bruhn-Olszewska et al., 2018; Fung et al., 2020), or are available commercially (e.g., Ihara et al., 2015; Scholtysek et al., 2024). Regardless of their origin, rigorous verification of purity and structural integrity is essential to ensure accurate and reproducible results. Impurities or degradation products can significantly impact detection efficiency, interfere with quantification, and lead to misleading conclusions, particularly in sensitive analytical techniques.

In summary, while chromatography and mass spectrometry-based approaches remain the gold standard for (p)ppGpp detection due to their sensitivity and quantitative capabilities, newer fluorescence-based and real-time detection methods offer promising advancements. Besides those described above, for example, a method employing Eu-MoS_2_ quantum dots relying on energy transfer effect was described to detect ppGpp (Rong et al., 2020). However, these methods require further optimization to overcome specificity and application constraints. As these novel approaches continue to evolve, they hold great potential to complement traditional techniques and drive further discoveries in bacterial signaling and adaptation mechanisms.
